# Comparing nanobody and aptamer-based capacitive sensing for detection of interleukin-6 (IL-6) at physiologically relevant levels

**DOI:** 10.1007/s00216-023-04973-4

**Published:** 2023-10-05

**Authors:** Raquel Sánchez-Salcedo, Rebeca Miranda-Castro, Noemí de-los-Santos-Álvarez, María Jesús Lobo-Castañón, Damion K. Corrigan

**Affiliations:** 1 Departamento de Química Física y Analítica, Universidad de Oviedo, Av. Julián Clavería 8, 33006 Oviedo, Spain; 2 Instituto de Investigación Sanitaria del Principado de Asturias, Av. de Roma, 33011 Oviedo, Spain; 3 Department of Pure & Applied Chemistry, University of Strathclyde, Thomas Graham Building, 295 Cathedral Street, Glasgow, G1 1XL UK

**Keywords:** Capacitive impedance spectroscopy, Electrochemical biosensing, Interleukin-6, Immunosensors, Aptasensors

## Abstract

A major societal challenge is the development of the necessary tools for early diagnosis of diseases such as cancer and sepsis. Consequently, there is a concerted push to develop low-cost and non-invasive methods of analysis with high sensitivity and selectivity. A notable trend is the development of highly sensitive methods that are not only amenable for point-of-care (*POC*) testing, but also for wearable devices allowing continuous monitoring of biomarkers. In this context, a non-invasive test for the detection of a promising biomarker, the protein Interleukin-6 (IL-6), could represent a significant advance in the clinical management of cancer, in monitoring the chemotherapy response, or for prompt diagnosis of sepsis. This work reports a capacitive electrochemical impedance spectroscopy sensing platform tailored towards *POC* detection and treatment monitoring in human serum. The specific recognition of IL-6 was achieved employing gold surfaces modified with an anti-IL6 nanobody (anti-IL-6 VHH) or a specific IL-6 aptamer. In the first system, the anti-IL-6 VHH was covalently attached to the gold surface using a binary self-assembled-monolayer (SAM) of 6-mercapto-1-hexanol (MCH) and 11-mercaptoundecanoic acid. In the second system, the aptamer was chemisorbed onto the surface in a mixed SAM layer with MCH. The analytical performance for each label-free sensor was evaluated in buffer and 10% human serum samples and then compared. The results of this work were generated using a low-cost, thin film eight-channel gold sensor array produced on a flexible substrate providing useful information on the future design of *POC* and wearable impedance biomarker detection platforms.

## Introduction

Currently, one of the major goals in healthcare research is the improvement of diagnosis, prognosis, and monitoring of diseases that have large social impact, often with poor patient outcomes such as cancer and sepsis. As a result of this, it is no surprising that development of efficient, high-performance analysis methods to improve existing diagnostic pathways and patient management systems is one of the main goals in the diagnostics research space. The need for efficient and early diagnosis has led researchers to evaluate different proteins as potential biomarkers for unregulated physiological states and a range of diseases. One promising category of biomarkers is the group of small and soluble proteins known as cytokines, which are released from cells of the immune system and influenced by acute inflammatory processes, infection, trauma, immune response, or the evolution of particular diseases [1]. Cytokines can act as pro- or anti-inflammatory molecules and play an important role in cell replication, in cell apoptosis, and in the repair of chemically induced tissue damage. Hence, in the biomedical field, cytokines are considered valuable markers for diagnosing disease or monitoring treatment response [2].

Among all cytokines, interleukin-6 or IL-6, a single chain phosphorylated 21-kDa glycoprotein composed of 184 amino acids [3] and one of the key pro-inflammatory proteins produced by many different cell types, including cells of the immune system, has been studied since its discovery in 1986 by Hirano et al. [4]. IL-6 can mediate both inflammatory and stress-induced responses. Moreover, it has been reported that elevated levels of this protein in the human body indicate activation of the cytokine pathway related to acute inflammatory processes (such as those seen in sepsis and brain injuries) or chronic activation leading to disease progression, including cancer or cardiological pathologies [5, 6]. In abnormal states, which are brought about by the inflammatory response, IL-6 levels in human fluids can rise into the range of 100 pg/mL to 1 ng/mL compared to the clinically normal levels in serum or blood of 5 pg/mL [7]. When specifically considering cancer, elevated IL-6 effects lead to proliferation, invasion, and metastasis of tumour cells, as well as suppression of antitumor immunity. Changes in IL-6 levels have been also observed after tumour removal surgery or during chemotherapy, which might be an indicator of therapy response. In addition, there is some evidence that suggests IL-6 concentration could be related to tumour size, recurrence, or disease stage [8].

It is therefore clear that the development of analytical methods capable of fast, reliable, specific, and highly sensitive determination of IL-6 concentration would be of great interest and represent a significant advance for the early diagnosis of a variety of conditions, including cancer, potentially leading to improved survival and outcome rates for patients. With this in mind, biosensors have gained popularity over the last 15 years due to their advantages of ease of handling, high analytical sensitivity, potential for multiple and parallel detection and connectedness [9], manufacturability, and suitability for POC use. Electrochemical biosensors represent a powerful strategy for low-cost, rapid, highly sensitive POC detection of clinically relevant markers [10, 11]. Furthermore, their applicability to liquid biopsy and adaptability to wearable devices make them particularly appealing [12, 13]. Among all electrochemical measurement techniques, electrochemical impedance spectroscopy (EIS) is the most sensitive with respect to surface-constrained processes, but compared to amperometric and potentiometric techniques has not yet led to as many applications in real-world use [14]. The EIS technique can be further distinguished into two modes of measurement: faradaic and non-faradaic mode. While faradaic measurements rely on the presence of a redox probe in solution, non-faradaic or capacitive mode gives the opportunity to perform reagentless, label-free, real-time, and non-invasive determination of receptor-ligand interactions [15]. Therefore, non-faradaic mode has the advantage of allowing direct measurements (e.g. in a wearable format) and overall a simplified practical implementation [14, 16]. However, the capacitive measurement mode has been less studied with fewer capacitive biosensors reported to date [17]. Beyond the measurement itself, a crucial aspect of developing a new electrochemical detection technology is the nature of the surface, the ionic strength of the media, and the choice of the bioreceptor for recognition of the target [18, 19]. Different natural and synthetic molecular recognition elements, such as antibodies [20], IL-6 receptors [21], aptamers [22], and molecularly imprinted polymers (MIPs) [23], have proven to be useful for the development of electrochemical biosensors [24], using various conductive substrates for their immobilization. In this study, we proposed the use of gold surfaces that contribute to the simplicity of functionalization and the attachment of the molecular receptors [25, 26]. In the field of clinical analysis, antibodies (Ab) are considered the gold standard for immunoassays and, as such, most of the IL-6 sensors developed so far are immunosensors. However, the variability between different batches, the difficult process and high costs of obtaining monoclonal Abs [27], and their large relative molecular weight serving as a limiting factor of sensitivity in impedance assays make it necessary to explore alternative bioreceptors.

Nanobodies (VHHs) and aptamers are receptor molecules that could replace the traditional Ab [28] as currently used in electrochemical POC technologies. Single-domain Abs called nanobodies (VHHs) are recombinant fragments of an approximate weight of 15 kDa containing the variable chain of a subtype of Ab that appear in camelids and do not have light chains. They have some advantages such as simple engineering, lower price production, better stability, high solubility, and the possibility to obtain higher density onto the surfaces and greater analytical signal gain. Their sequence can be transferred to bacterial expression systems for their production [29, 30]. Aptamers are synthetic single-stranded oligonucleotides which are currently gaining in popularity in bioanalysis because of advantages such as easy chemical manufacturing processes, thermal and chemical stability, and simple modification with different labels or marker molecules. They can be selected to recognize a wide range of targets including toxic compounds or non-immunogenic molecules [31, 32]. An in vitro process called SELEX (Systematic Evolution of Ligand by EXponential enrichment) is generally used for their selection. This process mimics the natural selection process [33, 34], leading to sequences capable of recognizing specific targets typically through a conformational change on binding [28, 35].

Another type of synthetic receptors for the efficient capture of IL-6 is MIPs, which are obtained by polymerization of a monomer in the presence of the target. After target removal, the specific binding sites are revealed. MIPs are resistant to harsh conditions, but the accessibility of the binding sites to proteins can be rather limited [23, 24].

**Fig. 1:**
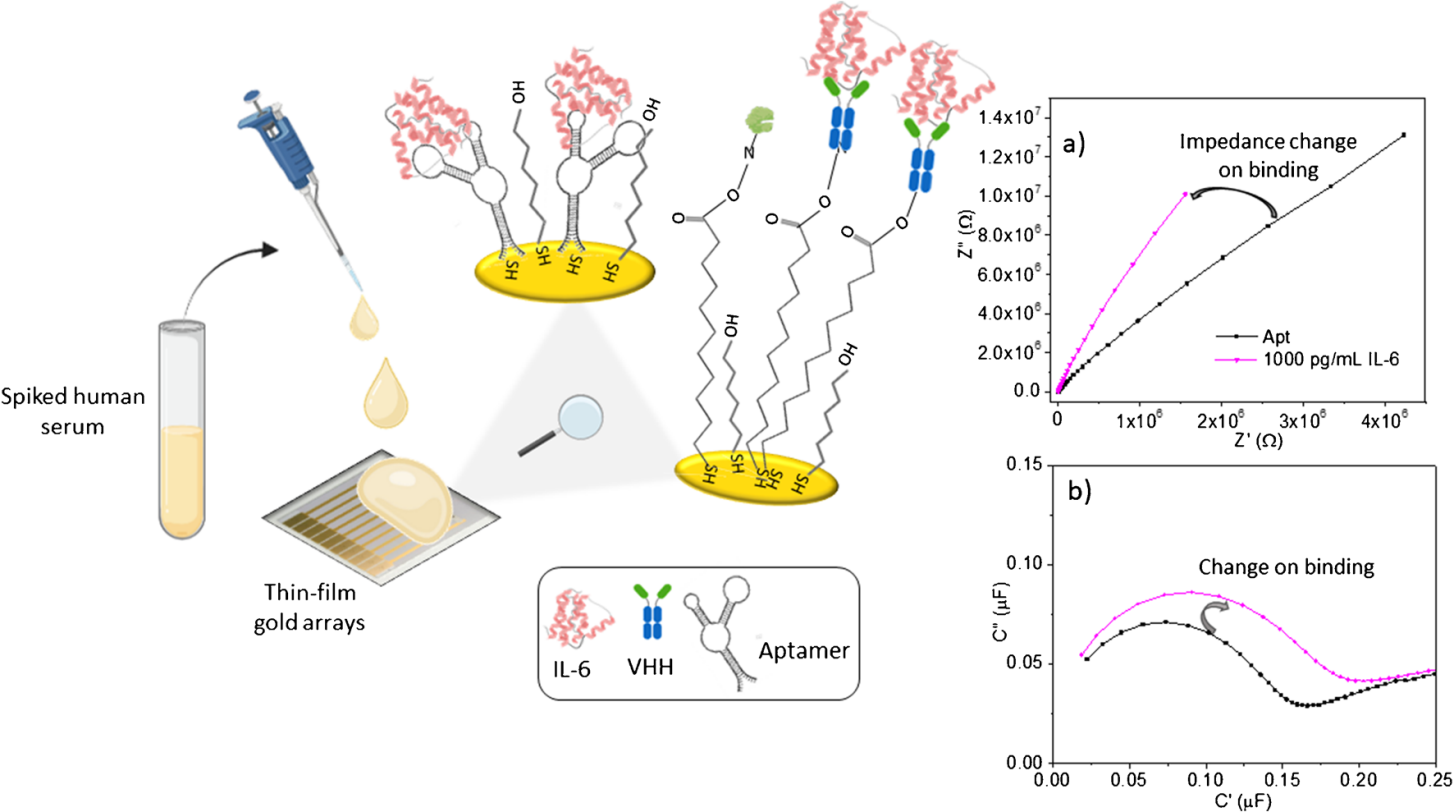
Schematic drawing of conceptual basis of the work. Gold electrodes are modified with a nanobody or an aptamer specifically recognizing IL-6. There is a change in the non-faradaic impedance spectra when these surfaces are challenged in serum samples containing IL-6, which can be observed in both impedance (**a**) and capacitance planes (**b**)

In this work, a low-cost, flexible, eight-channel gold biosensor chip was used to assess the relative performance of two sensing platforms for detection of IL-6 protein in both buffer and spiked 10% human serum. The first platform utilizes an anti-IL-6-VHH nanobody receptor and the second one employs an IL-6-aptamer, with both systems running in a label-free impedimetric format on the gold eight-channel multi-electrodes. The assay, which employs capacitive measurements in the absence of a soluble redox marker, is appealing because it makes the devices suitable for future use in a wearable device. The direct comparison of the nanobody and aptamer receptors is highly informative because it provides performance data on the analytical response of each receptor and again points to the best use case for eventual wearable use. The conceptual basis of the study is shown in Fig. 1.

## Materials and methods

### Reagents

N-(3-Dimethylaminopropyl)-N′-ethyl-carbodiimide hydrochloride (EDC), 6-mercapto-1-hexanol (MCH), N-hydroxysuccinimide (NHS), ethanolamine, 11-mercaptoundecanoic acid (MUA), Tris-(2-carboxyethyl)phosphine hydrochloride (TCEP), dithiothreitol (DTT), sodium acetate, C-reactive protein (ref 236,606), and human serum (H4522) were purchased from Sigma-Aldrich (Poole, Dorset). Potassium ferricyanide and potassium ferrocyanide were obtained from Fisher Scientific (Loughborough, UK). Phosphate buffer saline 10 × concentrate pH 7.4 (10 × PBS) and deionized water were purchased from Scientific Laboratory Supplies Limited (Nottingham, UK). Proteintech supplied the recombinant human IL-6 (HZ-1019) and the monoclonal single-domain antibody or nanobody (anti-IL-6 VHH) was supplied by QVQ (Utrecht, The Netherlands). Recombinant human MMP3 protein (513-MP) was acquired from R%D Systems. The aptamer was supplied by CreativeBiolabs (Shirley, NY, USA). As electrodes, surface flexible thin film gold arrays (TFGAs) were used and obtained from FlexMedical Solutions (Livingston, UK).

### Cleaning process

A Zepto plasma asher from Diener electronic was used to clean the TFGAs under an oxygen plasma flow of 0.7 mbar of pressure and 50% of power. For that, the electrodes were placed in the vacuum chamber and one pulse of 1 min was applied.

### Electrode functionalization

#### IL-6 VHH immunosensor

Prior to the modification of the surface, the specific IL-6 nanobody (VHH) was treated for 3 h at 37 °C in a thermal cycler from miniPCRbio™ with 2.5 molar excess of TCEP to reduce disulphide bonds of the cysteine amino acid.

Immediately after the oxygen plasma treatment, a 1:3 mixture of MUA and MCH was dropped to the TFGA surface for an overnight incubation at 4 °C in a water-saturated atmosphere. For that, stock solutions of 100 mM of MCH and 100 mM of MUA were first prepared in absolute ethanol, and then diluted to 1 mM in NaAc pH 5.5. After overnight incubation, the carboxylic groups of the SAM were activated with a mixture of 100 mM EDC/25 mM NHS in NaAc pH 5.5 for 30 min, and a solution of 30 µg/mL of IL-6-VHH in NaAc was then added to the surface for 30 min for covalent binding. The remaining unreacted carboxylic groups were blocked with 1 M of ethanolamine in 1 × PBS for 15 min. Volumes of 15 µL were used for covering the TGFA and incubations were performed at room temperature (RT) unless otherwise stated.

#### IL6 aptasensor

Before using the commercially supplied specific IL-6 aptamer, the oxidized disulphide form was chemically reduced with 125 µL of 0.1 M DTT for 2 h at RT protected from the light, and then purified by elution through a Sephadex G25 column (NAP-10, GE Healthcare). Afterwards, the cleaned TFGAs were incubated for 1 h at RT with 15 µL of a mix Aptamer:MCH (1:100) being the final thiol concentration 100 µM. The resulting binary SAM was additionally blocked by incubation with MCH 1 mM for 40 min at RT.

#### Assay protocol

The functionalised electrodes were incubated with solutions of IL-6 in 1 × PBS, concentrations ranging from 5 to 10,000 pg/mL, for 30 min at RT. The intended target was substituted by C-reactive protein (CRP) or metalloproteinase-3 (MMP-3) in the negative controls. Following each incubation and before the EIS measurement, a rinse with 1 × PBS was performed.

#### Faradaic and non-faradaic EIS measurements

TFGAs from FlexMedical, 8-gold sputtered working electrodes along with a common gold counter electrode and a Ag|AgCl printed common pseudo-reference electrode, were used to perform all the impedance measurements. The characterization of the functionalization of the electrode was carried out in faradaic mode in 5 mM [Fe(CN)_4_]^3−/4−^ in 1 × PBS and the measurements with the affinity sensors were performed in non-faradaic mode in 1 × PBS. EIS was measured against an open-circuit potential from 10 kHz to 0.1 Hz. *E*_ac_ (potential amplitude) was set at 0.01 V_rms_ and *E*_dc_ (potential during measurement) at 0 V. Fifty frequencies were recorded for all the experiments. A PalmSens4 potentiostat and PSTrace 5.9 software from Palmsens BV (Houten, The Netherlands) were used to perform all electrochemical measurements. Capacitance values were obtained for each frequency and subsequently analysed.

### Storage protocol

Once generated, the sensing layers were properly treated before being stored [36]. They were incubated with a solution of 2.5% of glucose and BSA as preservatives in 1 × PBS for 30 min at RT. Afterwards, they were dried under an argon stream and stored in Petri dishes in a dry atmosphere at 4 °C until 2 weeks. After the storage period, prior to being used, the electrodes were washed with 1 × PBS and then rehydrated with the same buffer for 2 h. The sensor surface stability was evaluated by measuring the capacitance of 50 pg/mL of IL6.

## Results and discussion

### Monitoring sensors fabrication

**Fig. 2:**
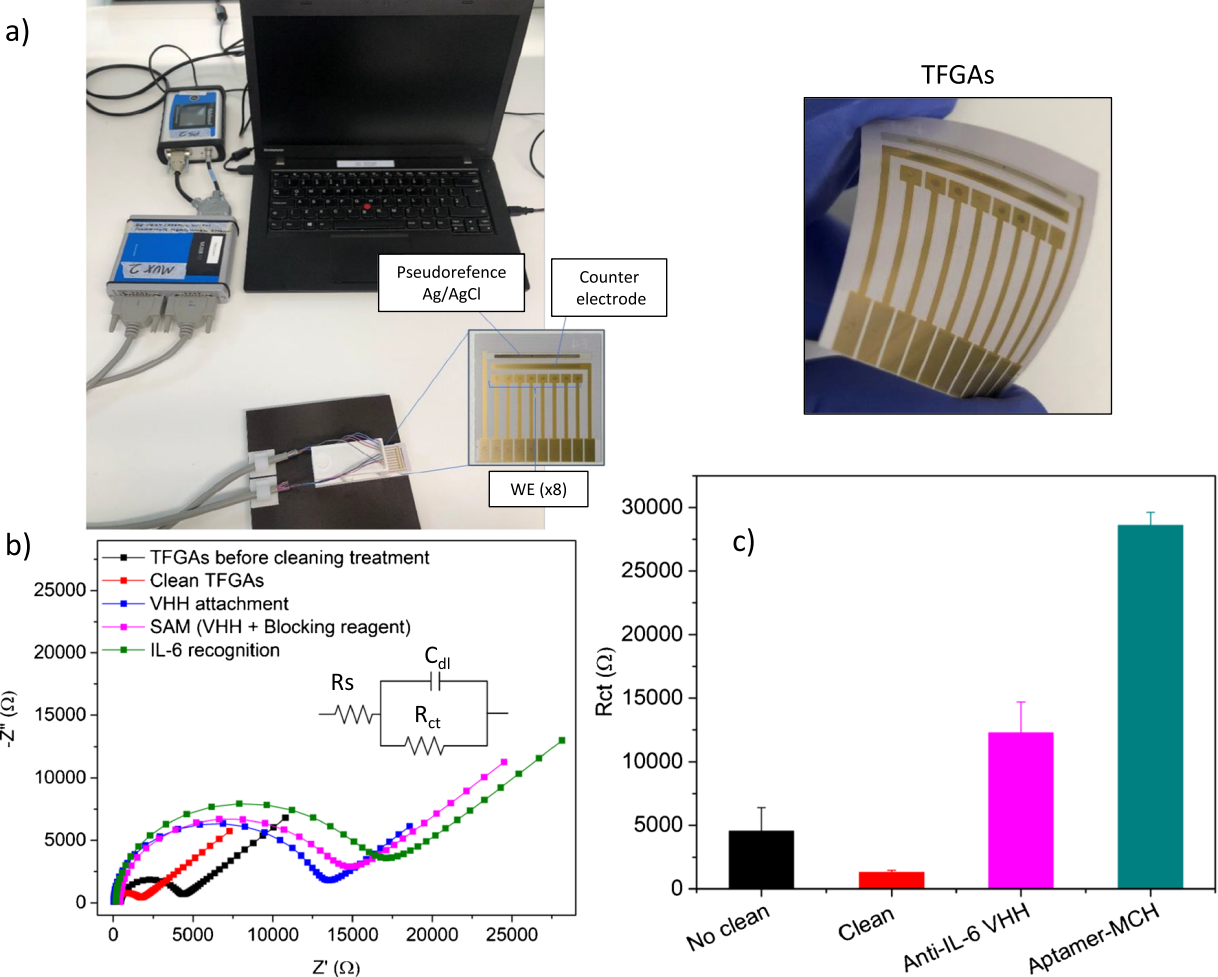
(**a**) TFGAs experimental setup including potentiostat, multichannel unit, bespoke connector, and TFGA chips. Characterization of functionalization steps of the immunosensor by faradaic EIS. (**b**) Nyquist plots at different modification steps and (**c**) charge transfer resistance for pre-cleaning, post-cleaning TGFAs, and for the VHH and aptamer modified sensing surfaces

The thin film gold arrays (TFGAs) contained eight 1-mm disc Au working electrodes with combined counter (Au) and reference electrode (Ag/AgCl) all designed to interface with a potentiostat and multiplexer unit (see Fig. 2a). The TFGA electrodes were subjected to an oxygen plasma treatment to obtain gold surfaces suitably clean for the subsequent formation of the sensing layers and attachment of bioreceptor probes. Faradaic EIS measurements were performed to verify electrode cleanliness and to sequentially monitor the functionalization process. To analyse the data, the Nyquist plots for the TFGAs at each stage were fitted to a standard Randles equivalent circuit, obtaining the charge-transfer resistance (*R*_ct_) from the semicircle diameter. Low *R*_ct_ values (1313 ± 143 Ω) for the bare electrode after the oxygen plasma treatment were observed, which indicated the redox couple could freely participate in electron transfer reactions, ensuring that the electrodes were appropriately clean prior to functionalization. Having established a cleaning process and benchmarked the impedance values associated with the cleaned state, the next aim was to characterize the formation of various receptor-alkanethiol SAM layers following functionalization.

Electrodes modified with the anti-IL-6 VHH, conjugated to the mixed SAM through EDC/NHS coupling, gave a marked increase in *R*_ct_, quantitatively seen as a percentage change of 702% with the clean electrodes having a mean *R*_ct_ of 11,380 ± 1100 Ω. That suggested [Fe(CN)6]^−3/4^ ions had reduced their ability to participate in electron transfer due to nanobody attachment. After blocking the free carboxylic groups of the SAM with ethanolamine, *R*_ct_ gave a similar value of 11,550 ± 1737 Ω (Fig. 2b, c). Afterwards, an increase in resistance was observed again for the recognition of the IL-6 protein (100 pg/mL) with a mean *R*_ct_ value of 13,930 ± 973 Ω, seen as a percentage change of 23.9%. Meanwhile, the starting *R*_ct_ measured for the aptasensor sensing platform was 28,614 ± 1006 Ω (Fig. 2c), increasing with the recognition of the protein to a mean *R*_ct_ value of 53,070 ± 1702 Ω which corresponds to a percentage change of 85.6%.

### Assay performance using the anti-IL-6 VHH

After characterizing the functionalization and sensor assembly steps with faradaic EIS as a benchmarking method, the focus of the study switched to non-faradaic measurements, recording the change in the capacitance due to binding between target and receptor. The rationale for focusing on capacitive measurements was that this approach negates the requirement for a redox mediator in solution to sustain the measurement like in faradaic mode. Demonstrating the sensitivity of the measurement in simple 1 × PBS is fairly representative of human interstitial fluid (ISF) [37, 38]. The applicability of the measurement in human serum samples is also shown because the development of a wearable IL-6 measurement is a key aim of this research.

There is no consensus in the method of presenting impedance data for capacitive measurements related to the different plots used. In this work, the impedance data in the absence of redox probe in solution was represented as the imaginary part of the capacitance *C*″ (µF) against the real part *C*′ (µF), leading to a complex-plane capacitance plot. In this plot, and assuming a blocking electrode, the *C*′ values at the minimum value of *C*″, corresponding roughly to the double-layer capacitance (*C*_dl_), were obtained as the final readout. These *C*_dl_ values were used to analyse the performance of the biosensors. For comparison, data were also analysed in terms of %∆*C* [(*C* − *C*_o_)/*C*_o_] × 100, where *C* and *C*_o_ represent the *C*_dl_ value after and before incubation with IL-6 solutions.

The first sensing strategy evaluated was the anti-IL-6 VHH-modified sensor. A SAM-based immunosensor was designed by following the well-established process of overnight assembling of a mixture of two alkanethiols on clean gold surfaces. In this approach, a mixture of 1 mM MUA:MCH (1:3) was used to form a well-ordered layer at the interface, which through the use of the EDC molecule, a zero-length cross-linker, allowed covalent binding between terminal carboxyl groups on the SAM layer and primary amines on the bioreceptor with a good orientation, increasing likelihood of receptor-target recognition. To improve the performance and efficiency of this coupling method, NHS was introduced to increase the stability of the active ester converting it to an active NHS-ester [39].

The *C*_dl_ value originated from the sensing layer increased significantly upon exposure to an IL-6 concentration of 1000 pg/mL for 30 min (from 0.189 ± 0.008 to 0.217 ± 0.006 µF). To assess the inter- and intra-chip reproducibility of the immunosensor, we analysed the response of three different TFGAs chips. Capacitive signal changes of similar magnitudes were obtained, leading to a 12.6% of RSD for inter-chip and a 7.2% for intra-chip measurements. Therefore, it was concluded that the reproducibility of the electrochemical surface was adequate and acceptable for further assay development activity.

**Fig. 3:**
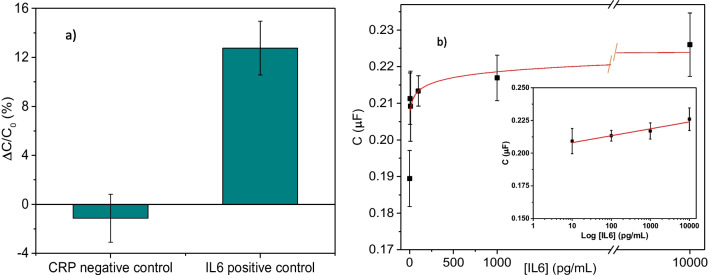
(**a**) Comparing positive (1000 pg/mL IL-6) and negative control (5700 pg/mL CRP) responses for the anti-IL-6 VHH. Error bars represent the standard deviation (SD) of 12 replicates (*n* = 12). (**b**) Dose–response curve for the IL-6 immunosensor in buffer. The inset represents the range of concentrations where the response is correlated with the concentration in a log scale. Error bars denote SD for *n* = 15

After verifying consistent behaviour and a positive response of the developed immunosensor, negative control experiments were performed by challenging the immunosensor with C-reactive protein (CRP), a protein that commonly appears in bodily fluids such as human serum, with increased abundance as a result of acute inflammation or tissue damage. Using 50 pM concentration for IL-6 and CRP (1000 pg/mL and 5700 pg/mL, respectively), all the negative control responses were found to be significantly lower compared to the positive signal. We observed a percentage of change with respect to the blank (∆*C* (%)) of − 1.1 ± 1.9 and 12.8 ± 2.2 for CRP and IL-6, respectively (Fig. 3a). Considering this evidence, it is concluded that IL-6 successfully and specifically bound to the anti-IL6 VHH while CRP did not, denoting not only the specificity of the VHH but also the effectiveness of the blocking process during sensor construction.

Once the efficiency and reproducibility of the functionalization of the electrodes and the specificity for IL-6 have been demonstrated, it is important to evaluate the dose–response effects. In these experiments, the capacitance consistently increased for additions of 5, 10, 100, 1000, and 10,000 pg/mL of IL-6, demonstrating a dose-dependent behaviour (Fig. 3b). The response of the immunosensor was fitted to a semi-logarithmic regression (*C* (µF) = (0.202 ± 0.003) + (0.005 ± 0.001) log [IL6] (pg/mL), *R* = 0.96) in the range between 10 pg/mL and 10 ng/mL.

Achieving good sensitivity is very important as the concentration of IL-6 in normal blood levels ranges from 2 to 10 pg/mL with elevations to 100–1000 pg/mL in the presence of trauma or pathological states [7]. With the designed immunosensor using the VHH, it is possible to achieve signals significantly different from the background for 10 pg/mL of IL6 which is in the pathological range. One important factor in impedimetric sensors is the fluctuation or change in the recorded capacitance, attributable to changes in the electrode surface or movement of ionic species in the measurement media, generating variations in double-layer capacitance [40, 41]. For this reason, a series of experiments designed to evaluate the signal drift were performed in buffer for the developed device. When the TFGAs were incubated in buffer over long periods of time, an obvious monotonic drift in the *C*_dl_ value was observed. Therefore, the specific response might be masked by the amplitude of this background drift, especially in a wearable format where long measurement times are employed. The large drift increase observed from PBS may be explained by a gradual reorganization of the SAM when applying the repetitive electrical inputs for EIS measurement. For this reason, we evaluate a new bioreceptor and strategy for the determination of this protein.

### Assay performance using the anti-IL-6 aptamer

We tested a label-free and reagentless impedimetric sensor targeting IL-6 employing a specific thiolated aptamer sequence as bioreceptor. Functionalization of the gold surface was possible using a simple chemisorption process from a mixed solution of Apt:MCH (1:100) with a final concentration of 100 µM MCH, allowing the formation of a strong bond to the gold surface as well as an organized monolayer with a defined (upright) orientation of the bioreceptor owing to the contribution of the MCH in SAM formation [42].

**Fig. 4:**
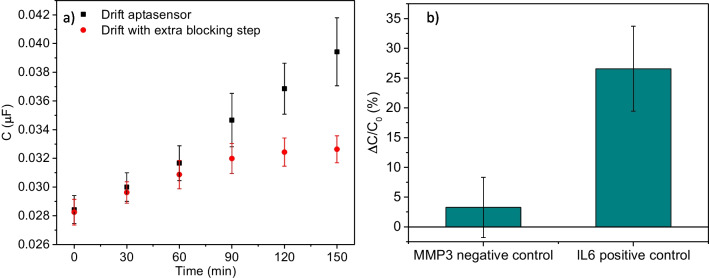
(**a**) Experiments evaluating the drift of the aptasensor in buffer before (black dots) and after an extra blocking step with 1 mM MCH (red dots). Error bars represent SD (*n* = 8). (**b**) Comparison between the percentage of change in the response for positive (1000 pg/mL IL-6) and negative control (2700 pg/mL MMP3) using the anti-IL-6 aptamer. Error bars represent the SD (*n* = 12)

As we highlighted, the changes in the signal in buffer over time had a large impact on the performance of the immunosensor. Therefore, an equivalent experiment was performed to first characterize the drift for the aptasensor. Moreover, to minimize the non-specific component of the response, additional experiments using an extra backfilling step consisting in the incubation of the sensing layer with MCH 1 mM for 40 min at RT were performed. The aptasensor exhibited a significantly lower baseline drift than the immunosensor. Over the course of 150 min of measurement in buffer, the capacitance arises from the aptasensor drift by 38%. This effect is further improved by the use of MCH in an extra backfilling step. The drift in this case is less than 14% after 90 min, and then, it leveled off (Fig. 4a). These results demonstrated that the aptasensor was less prone to baseline drift, outperforming the immunosensor in this regard. This may be explained by taking into account that the aptasensor showed higher initial charge transfer resistance values, pointing to the formation of a more ordered SAM layer that underwent lower levels of reorganization upon sensor electrification and repetitive EIS measurement.

To assess the response of the sensor after the recognition of IL-6 by the aptamer and to verify the selectivity of the aptamer, we conducted capacitive impedance measurements with the aptasensor. The TFGA electrodes were incubated with 1000 pg/mL of IL-6 for the positive control and with an equimolar concentration of MMP3 (2700 pg/mL) [43], a clinically relevant protein present in human fluids [38], for the negative control. We found a percentage of capacitance change of 3.3 ± 5.1 and 26.5 ± 7.1 for the negative and positive controls, respectively (Fig. 4b).

**Fig. 5:**
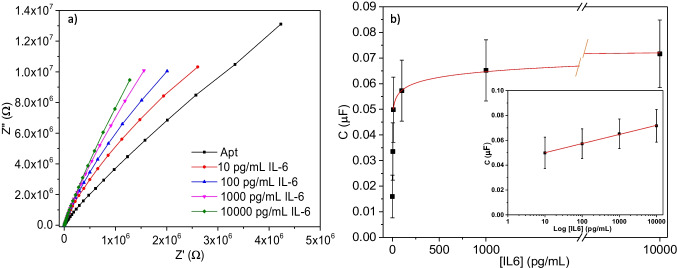
(**a**) Representative Nyquist plots for the aptasensor after challenging to increased concentrations of IL-6 in buffer. (**b**) Dose–response curve for the IL-6 aptasensor in buffer. The inset represents the range of concentrations where the response is correlated with the concentration in a log scale (*R* = 0.999) in a semi log scale (inset). Error bars represent SD for *n* = 15

Having achieved satisfactory selectivity and baseline drift, we next investigated the influence of IL-6 concentration on the response. Upon challenging the aptasensor in 1 × PBS buffer with increasing concentrations of IL-6 in the range of 10 to 10,000 pg/mL for 30 min at RT, the electrodes were washed, and the capacitive measurements were acquired. We obtained an increasing signal with a dose–response curve behaviour as shown in Fig. 5. The aptasensor dose–response curve fitted to a semi-logarithmic regression (*C* (µF) = (0.0427 ± 0.0006) + (0.0073 ± 0.0002) log[IL6] (pg/mL), *R* = 0.999) over the clinically relevant 10–10,000 pg/mL range.

### Evaluating aptasensor performance in complex sample

Once it was established that IL-6 could be detected with suitable sensitivity and selectivity in buffer using the IL-6 aptamer-based sensor, and keeping in mind that one of the goals of this research was to develop a device with POC testing application and compatible with wearable deployment, we next set out to characterize the performance of this device in complex media. Thus, the aptasensor was applied to IL-6 detection in filter-sterilized human serum samples spiked with 10,000 pg/mL IL-6. We recorded the device capacitive readout after challenging in the complex media (undiluted human serum) over 2 h, then adding 10,000 pg/mL IL-6. We obtained a slight change in the capacitance of 2.5 ± 0.4%, which is masked by the background drift. This presumably arises due to a hindered receptor-target interaction as a consequence of the high concentration of other proteins and the additional complexity of the medium. To further characterize the performance of the sensor, we performed the same experiment in 10% diluted human serum. In this medium, we observed a baseline drift similar to that in buffer, leading to a stabilization from 90 min, allowing to get a specific signal of IL-6 clearly different.

**Fig. 6:**
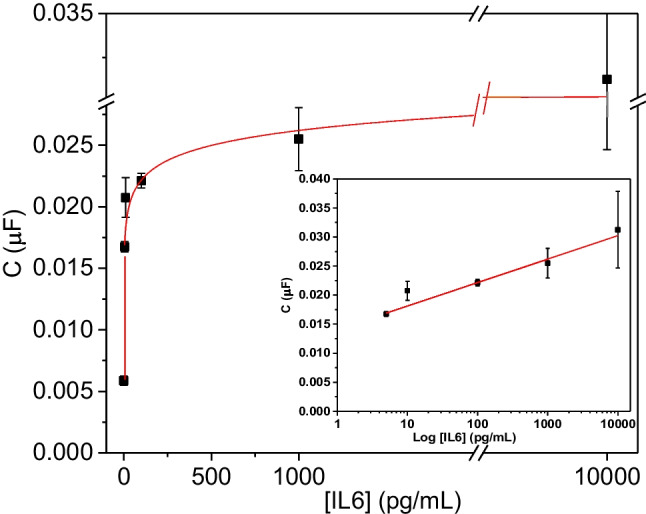
IL-6 dose–response curve obtained after subtracting the drift signal in 10% human serum (*R* = 0.98). Error bars represent SD (*n* = 8)

This finding prompted the construction of a dose–response curve with IL-6 spiked into 10% diluted human serum ranging from 5 to 10,000 pg/mL. Figure 6 shows the capacitive signal calculated after subtraction of the baseline drift in the same medium. A dose-dependent response is observed for IL-6, which fitted to a semi-logarithmic scale (*C* (µF) = (0.0141 ± 0.0007) + (0.0040 ± 0.0005) log [IL6] (pg/mL), *R* = 0.98) over the whole range of IL-6 concentrations tested.

Altogether, these results demonstrate that it was possible to construct sensitive biosensors for IL-6 using the TFGA chip. Being based on capacitive impedance measurements that do not require the presence of a soluble redox probe, the aptamer-based devices support specific signaling towards IL-6 in the range between 10 and 10,000 pg/mL and, unlike nanobody-based platform, the aptasensor was less influenced by the background drift, which becomes stable after 90 min. This is most likely due to a more tightly packed SAM layer (as evidenced by faradaic EIS experiments). Moreover, the aptasensor retained much of its response when challenged with complex samples (10% human serum). Comparable analytical performance to previously reported for microelectrode-based IL-6 immunosensors [1] as well as to some other electrochemical sensing platforms using antibodies as bioreceptor has been reached [24]. Besides, the developed aptasensor is competitive both in terms of sensitivity and detection range with the commercial methods used in clinical laboratories [3]. Although electrochemical biosensors for IL-6 with LODs lower than 1 pg/mL have been described [24], the results presented herein were generated using a low-cost eight-channel thin film gold capable of being produced in mass at ultra-low cost (< $ 2 US per strip). Future directions for this work include evaluation of sensor performance and stability in interstitial fluid (ISF) because we envisage skin-based testing of the immune component of ISF as a major area for wearable sensing and continuous monitoring in future years.

## Conclusions

In this work, a simple, label-free, capacitive-based method for the determination of IL-6, a protein biomarker with high relevance in the clinical field, is reported. The assay is implemented on a low-cost sensor array compatible with high-volume manufacturing techniques. We developed two systems, each using a different bioreceptor for the same biological target, an anti-IL-6 VHH and a thiolated IL-6 aptamer. When characterized by faradaic EIS, the expected chemical functionalization response and assay development were observed from the flexible TFGAs. Furthermore, they showed a good performance without a redox marker, thus pointing the way to the suitability and compatibility of the designs with POC or wearable devices. When evaluated in buffer, 10% human serum, human serum and with respect to baseline drift, the aptasensor was found to be the superior system. This is likely due to the formation of a more ordered and tightly packed SAM with this particular receptor and immobilization strategy, which also confers the advantages typically attributed to the aptamer-based devices such as low reagent costs, high-volume production potential, and good storage stability.

## References

[1] Oh C, Park B, Li C, Maldarelli C, Schaefer JL, Datta-Chaudhuri T, Bohn PW (2021). Electrochemical immunosensing of Interleukin-6 in human cerebrospinal fluid and human serum as an early biomarker for traumatic brain injury. ACS Meas Sci Au.

[2] Liu G, Qi M, Hutchinson MR, Yang G, Goldys EM (2016). Recent advances in cytokine detection by immunosensing. Biosens Bioelectron.

[3] Choi YJ, Roh J, Kim S, Lee KA, Park Y (2022). Comparison of IL-6 measurement methods with a special emphasis on COVID-19 patients according to equipment and sample type. J Clin Lab Anal.

[4] Hirano T, Yasukawa K, Harada H, Taga T, Watanabe Y, Matsuda T, Kashiwamura S, Nakajima K, Koyama K, Iwamatsu A, Tsunasawa S, Sakiyama F, Matsui H, Takahara Y, Taniguchi T, Kishimoto T (1986). Complementary DNA for a novel human interleukin (BSF-2) that induces B lymphocytes to produce immunoglobulin. Nature.

[5] Peppler WT, Townsend LK, Wright DC (2020). Recent advances in the role of interleukin-6 in health and disease. Curr Opin Pharmacol.

[6] Ehlers M, Grotzinger J, Dehon FD, Mullberg J, Brakenhoff JPJ, Liu J, Wollmer A, Rose-John S (1994). Identification of two novel regions of human IL-6 responsible for receptor binding and signal transduction. J Immunol.

[7] Russell C, Ward AC, Vezza V, Hoskisson P, Alcorn D, Steenson DP, Corrigan DK (2019). Development of a needle shaped microelectrode for electrochemical detection of the sepsis biomarker Interleukin-6 (IL-6) in real time. Biosens Bioelectron.

[8] Johnson DE, O’Keefe RA, Grandis JR (2018). Targeting the IL-6/JAK/STAT3 signalling axis in cancer. Nat Rev Clin Oncol.

[9] Ozkan-Ariksoysal D. Biosensors and their application in healthcare: Hot Topics. Future Medicine; 2013. pp 2–4. 10.4155/EBO.13.347.

[10] Takenaka S, Paleček E, Scheller F, Wang J (2005). Threading intercalators as redox indicators. Electrochemistry of nucleic acids and proteins.

[11] Choudhary M, Arora K. Electrochemical biosensors for early detection of cancer. In: Khan R, Parihar A, Sanghi SK, editors. Biosensor based advanced cancer diagnostics. Academic Press; 2022. pp. 123–151. 10.1016/B978-0-12-823424-2.00024-7.

[12] Attoye B, Pou C, Blair E, Rinaldi C, Thomson F, Baker MJ, Corrigan DK (2020). Developing a low-cost, simple-to-use electrochemical sensor for the detection of circulating tumour DNA in human fluids. Biosensors.

[13] Aydin EB, Aydin M, Sezgintürk MK. Advances in electrochemical immunosensors. In: Makowski GS, editor. Advances in Clinical Chemistry. 2019;92:1–57. 10.1016/bs.acc.2019.04.006. 10.1016/bs.acc.2019.04.006 31472751

[14] Bertok T, Lorencova L, Chocholova E, Jane E, Vikartovska A, Kasak P, Tkac J (2019). Electrochemical impedance spectroscopy based biosensors: mechanistic principles, analytical examples and challenges towards commercialization for assays of protein cancer biomarkers. ChemElectroChem.

[15] Quoc TV, Ngoc VN, Bui TT, Jen CP, Duc TC (2019). High-frequency interdigitated array electrode-based capacitive biosensor for protein detection. BioChip J.

[16] Lehr J, Fernandes FCB, Bueno PR, Davis JJ (2014). Label-free capacitive diagnostics: exploiting local redox probe state occupancy. Anal Chem.

[17] Díaz-Fernández A, Bernalte E, Fernández-Ramos C, Moise S, Estrela P, di Lorenzo M (2022). An impedimetric immunosensor for the selective detection of CD34+ T-cells in human serum. Sens Actuators B Chem.

[18] Mirsky VM, Riepl M, Wolfbeis OS (1997). Capacitive monitoring of protein immobilization and antigen-antibody reactions on monomolecular alkylthiol films on gold electrodes. Biosens Bioelectron.

[19] Weaver S, Mohammadi MH, Nakatsuka N (2023). Aptamer-functionalized capacitive biosensors. Biosens Bioelectron.

[20] Tanak AS, Muthukumar S, Krishnan S, Schully KL, Clark DV, Prasad S (2021). Multiplexed cytokine detection using electrochemical point-of-care sensing device towards rapid sepsis endotyping. Biosens Bioelectron..

[21] Aydın EB (2020). Highly sensitive impedimetric immunosensor for determination of interleukin 6 as a cancer biomarker by using conjugated polymer containing epoxy side groups modified disposable ITO electrode. Talanta.

[22] Gao Y, Nguyen DT, Yeo T, Lim SB, Tan WX, Madden LE, Jin L, Long JYK, Aloweni FAB, Liew YJA, Tan MLL, Ang SY, Maniya SD, Abdelwahab I, Loh KP, Chen C-H, Becker DL, Leavesley D, Ho JS, Lim CT (2021). A flexible multiplexed immunosensor for point-of-care in situ wound monitoring. Sci Adv..

[23] Yaman YT, Vural OA, Bolat G, Abaci S (2022). Peptide nanotubes/self-assembled polydopamine molecularly imprinted biochip for the impedimetric detection of human Interleukin-6. Bioelectrochem.

[24] McCrae LE, Ting W-T, Howlader MMR (2023). Advancing electrochemical biosensors for interleukin-6 detection. Biosens Bioelectron X.

[25] Wang X, Mei Z, Wang Y, Tang L (2017). Comparison of four methods for the biofunctionalization of gold nanorods by the introduction of sulfhydryl groups to antibodies. Beilstein Nanotechnol.

[26] Simões B, Guedens WJ, Keene C, Kubiak-Ossowska K, Mulheran P, Kotowska AM, Scurr DJ, Alexander MR, Broisat A, Johnson S, Muyldermans S, Devoogdt N, Adriaensens P, Mendes PM (2021). Direct immobilization of engineered nanobodies on gold sensors. ACS Appl Mater Interfaces.

[27] Baker M (2015). Reproducibility crisis: blame on the antibodies. Nature.

[28] Arshavsky-Graham S, Heuer C, Jiang X, Segal E (2022). Aptasensors versus immunosensors: Which will prevail?. Eng Life Sci.

[29] Scarrone M, González-Techera A, Alvez-Rosado R, Delfin-Riela T, Modernell Á, González-Sapienza G, Lassabe G (2021). Development of anti-human IgM nanobodies as universal reagents for general immunodiagnostics. N Biotechnol.

[30] Oloketuyi S, Bernedo R, Christmann A, Borkowska J, Cazzaniga G, Schuchmann HW, Niedziółka-Jönsson J, Szot-Karpińska K, Kolmar H, de Marco A (2021). Native Llama Nanobody Library Panning Performed by Phage and Yeast Display Provides Binders Suitable for C-Reactive Protein Detection. Biosensors.

[31] Sun H, Zhu X, Lu PY, Rosato RR, Tan W, Zu Y (2014). Oligonucleotide Aptamers: New Tools for Targeted Cancer Therapy. Mol Ther Nucleic Acids..

[32] Díaz-Fernández A, Lorenzo-Gómez R, Miranda-Castro R, de-los-Santos-Álvarez N, Lobo-Castañón MJ (2020). Electrochemical Aptasensors for Cancer Diagnosis in Biological Fluids – A Review. Anal Chim Acta..

[33] Ellington AD, Szostak JW (1990). In Vitro Selection of RNA Molecules That Bind Specific Ligands. Nature.

[34] Tuerk C, Gold L (1990). Systematic Evolution of Ligands by Exponential Enrichment: RNA Ligands to Bacteriophage T4 DNA Polymerase. Science.

[35] McConnell EM, Nguyen J, Li Y (2020). Aptamer-Based Biosensors for Environmental Monitoring. Front Chem.

[36] Miranda-Castro R, Sánchez-Salcedo R, Suárez-Álvarez B, de-los-Santos-Álvarez N, Miranda-Ordieres AJ, Lobo-Castañón MJ (2017). Thioaromatic DNA monolayers for target-amplification-free electrochemical sensing of environmental pathogenic bacteria. Biosens Bioelectron..

[37] Biosolve BV web. https://shop.biosolve-chemicals.eu/detail.php?id=2093. Accessed 9 Jul 2023.

[38] I*t’Is Foundation.* https://itis.swiss/virtual-population/tissue-properties/database/low-frequency-conductivity/. Accessed 14 Sept 2023.

[39] Hermanson, GT. Chapter 3: The Reactions of Bioconjugation. Bioconjugate Techniques Academic Press; 2013. pp. 229–258. 10.1016/b978-0-12-382239-0.00003-0.

[40] Kanyong P, Davis JJ (2020). Homogeneous functional self-assembled monolayers: faradaic impedance baseline signal drift suppression for high-sensitivity immunosensing of C-reactive. J Electroanal Chem.

[41] Riepl M, Mirsky V, Novotny I, Tvarozek V, Rehacek V, Wolfbeis S (1999). Optimization of Capacitive Affinity Sensors: Drift Suppression and Signal Amplification. Anal Chim Acta.

[42] Yourdshahyan Y, Zhang HK, Rappe AMN (2001). Alkyl thiol head-group interactions with the Au(111) surface. Phys Rev B.

[43] He D, Zhu Q, Zhou Q, Qi Q, Sun H, Zachariah LM, Wang G, Reveille JD, Guan Y, Zhou X (2017). Correlation of serum MMP3 and other biomarkers with clinical outcomes in patients with ankylosing spondylitis: A pilot study. Clin Rheumatol.

